# Effects of Yeast Protein–Whey Protein Composite Supplementation on Recovery from Exercise-Induced Muscle Damage in Well-Trained Athletes: A Randomized Controlled Trial

**DOI:** 10.3390/nu18203358

**Published:** 2026-10-14

**Authors:** Chunyan Xu, Lijing Gong, Zhen Ni, Xintang Wang

**Affiliations:** 1 Department of Sport Science Institute, Beijing Sport University, Beijing 100084, China; xucy@bsu.edu.cn; 2 Beijing Higher School Engineering Research Center of Sport Nutrition, Beijing Sport University, Beijing 100084, China; 3 Laboratory of Sports Stress and Adaptation of General Administration of Sport, Beijing Sport University, Beijing 100084, China; 4 China Institute of Sport and Health Science, Beijing Sport University, Beijing 100084, China; lijing.gong@bsu.edu.cn (L.G.); nizhen888@bsu.edu.cn (Z.N.); 5 Key Laboratory for Performance Training & Recovery of General Administration of Sport, Beijing 100084, China

**Keywords:** yeast protein, whey protein, exercise-induced muscle damage, creatine kinase, muscle soreness, muscle function, athletes

## Abstract

**Objectives:** Exercise-induced muscle damage (EIMD) can impair muscle function and subsequent athletic performance. This study examined whether an isonitrogenous yeast protein–whey protein composite (YP) modifies recovery from EIMD compared with whey protein (WP) and a water-only control (C) in well-trained athletes. **Methods:** In this prospective, randomized, three-arm trial, 54 athletes were allocated to YP, WP, or C; 48 completed all follow-up procedures. YP and WP provided 0.9 g·kg^−1^·day^−1^ of actual protein, whereas C received water only and was not energy or sensory matched to the protein interventions. After 7 days of supplementation, participants completed a bilateral isokinetic EIMD protocol; supplementation continued through the 48 h assessment. Outcomes were assessed before EIMD, immediately afterward, and at 24, 48, and 72 h and were organized into muscle damage and soreness, inflammatory responses, and muscle function and performance. Female and male athletes were analyzed separately using linear mixed-effects models with multiplicity-adjusted contrasts. The study was not powered to formally compare intervention effects between sexes. **Results:** EIMD produced time-dependent biochemical, perceptual, and functional changes. Holm-adjusted comparisons showed lower CK in YP than C at 24 h in females and 48 h in males. The female 24 h CK finding was considered exploratory because the corresponding group × time interaction did not remain significant after damage-domain BH-FDR correction; the immediate male YP–C difference was significant only in the log-scale analysis. In males, YP was associated with lower soreness averaged across assessments and a lower immediate post-EIMD IL-6 value than C. Selected male CMJ comparisons favored YP but were exploratory after broader multiplicity correction. Acute-decline analyses showed smaller losses with YP in bilateral MVC and right-leg eccentric peak torque in males; only the right-leg eccentric YP–C comparison remained significant after BH-FDR correction. **Conclusions:** Compared with water alone, YP favorably influenced selected biochemical, perceptual, and muscle-function responses following EIMD, with the clearest pattern observed in the male analysis. Its overall response pattern was broadly aligned with WP, supporting the recovery-related potential and practical value of incorporating yeast-derived protein into a whey-based formulation.

## 1. Introduction

Strenuous or unfamiliar exercise, particularly exercise involving repeated eccentric contractions, can induce exercise-induced muscle damage (EIMD), characterized by ultrastructural muscle alterations, efflux of myocellular proteins into the circulation, muscle soreness, inflammatory responses, and temporary reductions in muscle strength and power [1,2]. These responses may persist for several days and can impair readiness for subsequent training or competition [3,4]. The magnitude and time course of EIMD are influenced by the characteristics of the damaging exercise and individual factors, including training status, sex, nutritional status, and familiarity with the exercise task [5,6]. Because biochemical, perceptual, inflammatory, and functional measures reflect different aspects of EIMD and may follow distinct temporal patterns, these outcomes should be interpreted collectively rather than as interchangeable indicators [7,8].

Dietary protein is commonly used to support post-exercise remodeling and recovery. Exercising individuals are generally advised to consume approximately 1.4–2.0 g·kg^−1^·day^−1^, distributed across the day in adequate per-meal doses [9,10]. Whey protein is widely used because of its rapid digestion and high indispensable-amino-acid and leucine contents [11]. However, evidence that protein supplementation consistently reduces EIMD remains mixed. Meta-analyses suggest possible benefits for later CK responses and selected measures of muscle function, whereas effects on soreness and performance are less consistent [12,13,14]. In a three-group Nutrients trial using 0.9 g·kg^−1^·day^−1^, whey protein attenuated later circulating muscle-damage biomarkers relative to water, while pea protein showed an intermediate response and neither protein significantly altered soreness or fitness-test performance [15].

The increasing demand for alternative proteins has stimulated interest in microbial protein sources. Yeast-derived ingredients can provide a high proportion of protein with a relatively balanced amino-acid profile, while fermentation-based production can reduce dependence on livestock-derived raw materials and conventional agricultural production [16,17]. The nutritional quality, digestibility, and techno-functional properties of yeast-derived protein depend on the yeast strain and on processing steps such as cell-wall disruption, protein extraction, nucleic-acid reduction, and final product formulation [16,17,18]. However, yeast-derived protein can exhibit distinctive aroma and flavor attributes that may limit sensory acceptability when used at high inclusion levels [17,19]. Blending yeast-derived protein with whey protein may therefore represent a practical formulation strategy that increases the contribution of microbial protein while retaining a substantial whey-protein component. Nevertheless, direct evidence regarding the effects of yeast protein as a dietary protein source on recovery from EIMD remains scarce.

The present study compared an isonitrogenous yeast protein–whey protein composite (YP), whey protein alone (WP), and a water-only control (C) during recovery from a standardized bilateral EIMD protocol in well-trained female and male athletes. Outcomes were organized into three complementary domains: muscle damage and soreness, inflammatory responses, and muscle function and performance. We hypothesized that both protein-containing interventions would attenuate selected EIMD responses relative to water. Direct comparisons between YP and WP were conducted to explore whether replacing part of the whey protein with yeast-derived protein materially altered the observed recovery responses.

## 2. Materials and Methods

### 2.1. Study Design, Ethics, and Registration

This prospective, randomized, controlled, three-arm, parallel-group trial was conducted in accordance with the Declaration of Helsinki and was approved by the Ethics Committee of Beijing Sport University (approval no. 2024089H, 20 March 2024). All participants provided written informed consent before baseline assessment and any study-specific procedure. The trial was prospectively registered in the Chinese Clinical Trial Registry on 30 September 2024 (ChiCTR2400090476, https://www.chictr.org.cn/). Participants were recruited from 12 October 2024, and all intervention and follow-up procedures were completed by the end of December 2024 at Beijing Sport University, Beijing, China. Participants and members of the public were not involved in the design, conduct, interpretation, or reporting of the study.

### 2.2. Participants and Sample Size

Fifty-four competitive athletes were recruited from basketball, football, volleyball, martial arts, track and field, and ice sports. Participants were well-trained competitive athletes officially classified in China as first-class athletes or master athletes. Eligible participants were apparently healthy, had no condition expected to interfere with the intervention or outcome assessments, had not used protein, anti-inflammatory, or antioxidant supplements during the preceding 3 months, did not smoke or consume alcohol, and did not use analgesics or other substances known to affect metabolism.

Sample size was estimated using G*Power version 3.1.9.6. Assuming an effect size of f = 0.30, a two-sided α level of 0.05, and 80% power, the estimated minimum sample size was 45. Allowing for approximately 20% attrition, 54 athletes were recruited. Given the multidimensional nature of EIMD, outcomes were organized into three prespecified conceptual domains: muscle damage and soreness, inflammatory responses, and muscle function and performance. The sample-size calculation was based on the total study population rather than on separate sex-specific estimates. Female and male athletes were analyzed separately according to the prespecified analytical approach, allowing intervention effects to be evaluated within each sex. Given the modest subgroup sizes, the study was not designed to formally compare intervention effects between sexes.

### 2.3. Randomization, Allocation Concealment, and Blinding

After baseline assessment, an independent researcher generated sex-stratified random sequences using Microsoft Excel and allocated participants in a 1:1:1 ratio to YP, WP, or C. The allocation sequence was concealed from personnel responsible for enrollment and outcome assessment until assignment. Because C received water only, participant blinding across all three groups was not possible. The YP and WP formulations were pilot-tested and prepared to be as similar as practicable in packaging, color, odor, taste, and texture and were coded as A or B by an independent supplier. Participants in these groups and personnel handling the supplements were unaware of formulation identity. Participants were not formally asked to guess their allocation, and the success of blinding between YP and WP was not formally evaluated. Outcome assessors and data-analysis personnel remained blinded to group allocation until completion of the prespecified analyses.

### 2.4. Protein Supplementation and Dietary Assessment

Participants in YP and WP consumed three daily servings, each providing 0.3 g protein·kg^−1^ body mass in 250 mL of water, corresponding to a total supplemental protein dose of 0.9 g·kg^−1^·day^−1^. Participants in C consumed 250 mL of water at the corresponding administration times and received no protein supplement or energy- or sensory-matched placebo.

The YP formulation comprised 40% yeast protein ingredient (AnPro; Angel Yeast Co., Ltd., Yichang, Hubei, China; 80% protein), 40% whey protein isolate (Glanbia Nutritionals, Inc., Twin Falls, ID, USA; 90% protein), and 20% flavoring agents and excipients by powder mass. The WP formulation comprised 80% whey protein isolate (Glanbia Nutritionals, Inc., Twin Falls, ID, USA; 90% protein) and 20% flavoring agents and excipients. According to manufacturer-provided finished-product analyses, YP and WP contained 68.5 and 68.9 g protein, 4.3 and 5.6 g fat, 21.2 and 18.0 g carbohydrate, and 1685 and 1690 kJ energy per 100 g powder, respectively. YP contained 0.8 g nucleic acid per 100 g powder, whereas nucleic acid was not detected in WP. The indispensable-amino-acid profiles and complete composition data are provided in Table S1. To provide 0.3 g protein·kg^−1^ per serving, the powder doses were approximately 0.438 g·kg^−1^ for YP and 0.435 g·kg^−1^ for WP. Based on the listed energy contents, the calculated daily energy provision was approximately 22.1 kJ·kg^−1^·day^−1^ in both protein groups. Thus, the two protein interventions were isonitrogenous and closely matched in calculated energy delivery.

Supplementation was administered for 10 consecutive days. Participants completed 7 days of supplementation before undergoing the EIMD protocol on day 8. Supplementation continued on the EIMD day and throughout the 24 and 48 h recovery periods, with the 48 h assessment occurring on the final supplementation day. The 72 h assessment was conducted on day 11, after supplementation had ceased. On days without outcome assessments, the daily dose was divided into morning, midday, and evening servings. The morning serving was provided on the preceding day for self-administration, whereas the midday and evening servings were distributed and consumed under research-team supervision. On assessment days, no supplement was consumed before testing; the next scheduled serving was consumed with the first meal after all measurements had been completed. Participants were instructed to report any missed or incomplete self-administered serving.

Habitual dietary intake was assessed before supplementation using three 24 h dietary recalls covering two weekdays and one weekend or non-training day. Dietary records were analyzed using the Athlete and General-Population Dietary Nutrition Analysis and Management System developed by the Sports Nutrition Research Center, Institute of Sports Medicine, General Administration of Sport of China. Group- and sex-specific energy intake, absolute protein intake, and body-mass-normalized protein intake are reported in Table 1. Habitual protein intake averaged approximately 1.2 g·kg^−1^·day^−1^; when combined with supplementation, estimated total protein intake in YP and WP was approximately 2.1 g·kg^−1^·day^−1^.

Participants were instructed to maintain their habitual diet and training practices, avoid additional protein supplements or new recovery interventions, and refrain from strenuous lower-body exercise during the 72 h follow-up period. At each study visit, participants were asked about new or worsening symptoms, including gastrointestinal discomfort, allergic reactions, and other health problems. Throughout supplementation and follow-up, participants photographed their meals and submitted the images to the research team for qualitative monitoring of dietary adherence. Dietary intake was not standardized, and the photographs were not used for repeat quantitative nutrient analysis.

### 2.5. Exercise-Induced Muscle Damage Protocol

After a standardized warm-up, participants performed a bilateral knee flexion–extension protocol on an Isomed 2000 isokinetic dynamometer (D&R Ferstl GmbH, Hemau, Germany), adapted from previously described eccentric and isokinetic EIMD protocols [20,21]. The right leg was exercised before the left leg in all participants. For each leg, the range of motion was set from 90° to 150° according to the dynamometer angle convention, corresponding to an angular displacement of 60°. Participants performed maximal voluntary contractions during both the concentric and eccentric phases with real-time visual feedback and standardized verbal encouragement.

Each leg completed a minimum of 10 sets of 10 repetitions, with 30 s of rest between sets [20]. Participants who had not reached the prespecified fatigue threshold after 10 sets continued until the threshold was met, consistent with the exhaustion-based approach used in previous isokinetic EIMD models [21]. Fatigue was operationally defined as five consecutive repetitions during which both concentric and eccentric peak torque fell below 60% of the initial peak torque recorded for the corresponding contraction mode and leg. A decline to 60% of maximum torque has previously been used as a task-termination threshold during sustained maximal contractions [22]. The number of completed sets was recorded separately for each leg, and total repetitions were calculated as completed sets × 10. Sex- and group-specific summaries are reported in Table S2. Total mechanical work and time under tension were not recorded.

### 2.6. Outcome Assessments

In accordance with the trial-registration record, the primary outcomes reported in this article were serum CK, circulating cytokines (IL-6, IL-1β, TNF-α, and IL-10), CMJ performance, MVC, and isokinetic concentric and eccentric peak torque. Perceived muscle soreness assessed using the VAS was the secondary outcome. These outcomes were assessed before EIMD, immediately after EIMD, and at 24, 48, and 72 h after EIMD, as described below. Additional registered outcomes, including metabolomic analyses, addressed separate mechanistic objectives and are outside the scope of the present report. The registration did not designate a single primary biochemical endpoint or a single primary functional endpoint; therefore, no outcome was retrospectively promoted to a sole confirmatory endpoint.

#### 2.6.1. Blood Sampling and Biochemical Analyses

Fasting venous blood was collected at pre-EIMD baseline, immediately after EIMD, and 24, 48, and 72 h after EIMD. Samples were allowed to clot for 1 h at room temperature, centrifuged at 3000 rpm for 10 min at 4 °C, aliquoted, and stored until analysis. Serum CK, IL-6, TNF-α, IL-10, and IL-1β were quantified. IL-6 was measured using the Access IL-6 chemiluminescent immunoassay (catalog no. A16369; Beckman Coulter, Brea, CA, USA). TNF-α, IL-10, and IL-1β were measured using chemiluminescent immunoassay kits supplied by Sichuan Orienter Bioengineering Co., Ltd. (Chengdu, China). Analyses were performed using a UniCel DxI 800 automated chemiluminescent immunoassay analyzer (Beckman Coulter).

#### 2.6.2. Muscle Soreness

Perceived quadriceps soreness was evaluated with a 100 mm visual analog scale (VAS) at all five assessments. Participants sat on the same chair and relaxed before standing naturally without external assistance. They then rated the soreness perceived in the quadriceps during the sit-to-stand transition and quiet standing. The scale was anchored by “no soreness” at 0 mm and “worst imaginable soreness” at 100 mm.

#### 2.6.3. Countermovement Jump

CMJ performance was assessed using a Kunwei vertical-jump force platform and its associated software (Kunwei, China). Participants stood with their feet approximately shoulder-width apart and kept their hands fixed on their hips throughout the movement; arm swing was not permitted. After a rapid downward countermovement, participants immediately performed a maximal vertical jump and landed on the platform in approximately the same position. Two familiarization trials were followed by three maximal recorded trials separated by 1 min, and the highest valid jump height exported by the manufacturer software was retained.

#### 2.6.4. Maximal Voluntary Isometric Contraction and Isokinetic Peak Torque

Quadriceps MVC and concentric and eccentric peak torque were assessed using the Isomed 2000 dynamometer. For MVC, the knee was fixed at 60° of flexion. Participants performed three 5 s maximal knee-extension efforts separated by 90 s, and the highest value was retained. Concentric and eccentric quadriceps peak torque were assessed separately for each leg at 60°·s^−1^ through the standardized 60° range of motion. Participants completed 10 maximal repetitions, and the highest peak torque was used. The right leg was assessed before the left leg for all participants. Limb dominance was not recorded.

### 2.7. Statistical Analysis

Analyses included the 48 participants who completed follow-up; the six participants who withdrew had no post-randomization outcome data. All available measurements from completers were retained without imputation. Female and male athletes were analyzed separately because several muscle-damage and muscle-function outcomes differed substantially in magnitude and variability by sex. For each outcome, baseline, immediately post-EIMD, and 24, 48, and 72 h post-EIMD were treated as five levels of a categorical time factor. Linear mixed-effects models included group, time, and group × time as fixed effects and participant as a random intercept.

The group × time interaction was evaluated first. Significant interactions were followed by group comparisons at each time point. When the interaction was not significant, an additive model was used to estimate the main effects of group and time. Group main effects represented differences averaged across all five assessments and were not interpreted as evidence of different recovery rates. Pairwise comparisons among YP, WP, and C were adjusted using the Holm procedure within each prespecified contrast family. An intention-to-treat analysis was not prespecified. Because all six withdrawals occurred before any post-randomization outcome was obtained, these participants could not contribute information to the longitudinal models, and no outcome imputation was performed. Sex was not included as an additional interaction term because separate sex-stratified models were fitted, and no formal group × time × sex comparison was performed. Findings were interpreted within each sex, and differences in statistical significance between female and male analyses were not considered evidence of sex-specific treatment effects.

In secondary muscle-function analyses, acute decline was calculated as baseline minus immediately post-EIMD for CMJ, MVC, and concentric and eccentric peak torque, with larger positive values indicating greater functional loss. Sex-stratified one-way ANOVA was used to compare groups, followed by Fisher’s protected least significant difference test only when the omnibus test was significant. Changes from immediately post-EIMD to 24, 48, and 72 h were analyzed analogously as exploratory recovery outcomes. Homogeneity of variance was assessed using Levene’s test, and Welch’s ANOVA was used as a sensitivity analysis when this assumption was violated. Mean differences, 95% CIs, *p* values, and Hedges’ g were reported.

CK and cytokine concentrations were natural-log transformed because of their right-skewed distributions; back-transformed estimates are reported as geometric means or ratios. CK was additionally analyzed on its original scale as a sensitivity analysis. VAS, CMJ, MVC, and isokinetic peak torque were analyzed on their recorded scales; CMJ was expressed relative to baseline (100%). Statistical significance was set at *p* < 0.05. As a broader multiplicity sensitivity analysis, the Benjamini–Hochberg false-discovery-rate procedure was applied across related outcomes within each conceptual domain and sex and, for change-score analyses, within each sex and interval. Findings that remained significant after the broader BH-FDR correction were considered to have stronger multiplicity-controlled support; findings significant only in the outcome-specific analyses were interpreted as exploratory. No result was described as formally confirmatory evidence for a single endpoint. Statistical analyses were performed in Python 3.12.10 using statsmodels 0.14.5, SciPy 1.16.1, pandas 3.0.5, and NumPy 2.5.2. Figures were generated using Matplotlib 3.10.5. Completed EIMD set counts were compared among groups within each sex using Kruskal–Wallis tests.

## 3. Results

### 3.1. Participant Flow and Intervention Adherence

**Figure 1:**
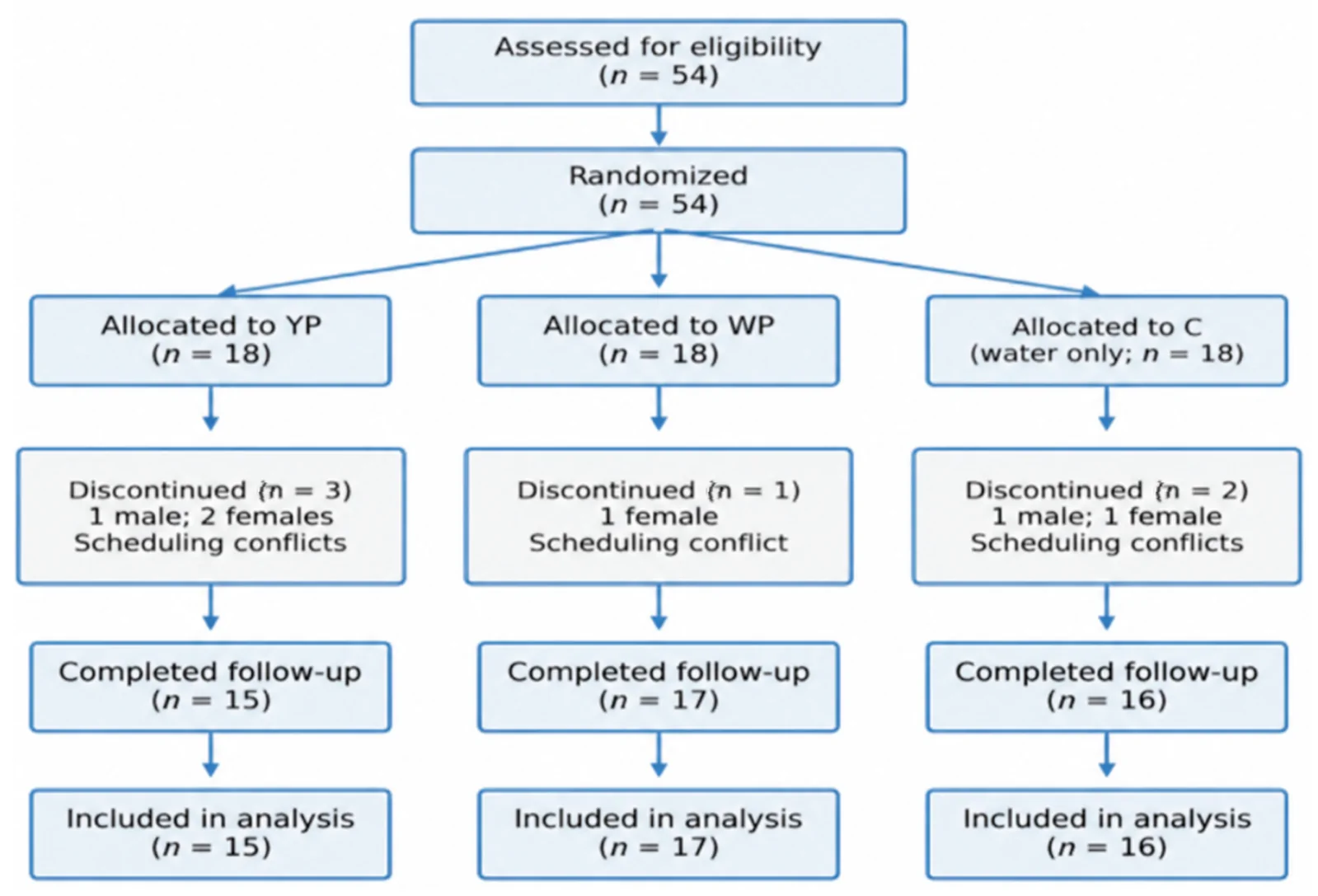
CONSORT participant-flow diagram. All discontinuations were attributed to scheduling conflicts. YP, yeast protein–whey protein composite; WP, whey protein; C, water-only control.

Fifty-four athletes were randomized, with 18 assigned to each group. Six discontinued because of scheduling conflicts unrelated to the intervention: three in YP (one male and two females), one female in WP, and two in C (one male and one female). None provided post-randomization outcome data. The sex-specific group sizes ranged from seven to nine participants and were 7 females/8 males in YP, 8/9 in WP, and 8/8 in C (Figure 1). All participants included in the analyses completed their assigned intervention and follow-up procedures, and no major deviations from the supplementation protocol were documented. No adverse events were reported in any group. Expected transient muscle soreness following the EIMD protocol was assessed as a study outcome and was not classified as an adverse event. Baseline participant characteristics and habitual dietary intake are summarized in Table 1; outcome-specific sample sizes are reported in Table S3. The total number of completed EIMD sets did not differ among groups in females (Kruskal–Wallis *p* = 0.182) or males (*p* = 0.425); descriptive set and repetition counts are presented in Table S2. The distribution of sport disciplines across groups is provided in Table S4. Volleyball was the largest discipline in each group, whereas several other disciplines were represented by only one or two athletes.

### 3.2. Muscle Damage and Soreness

CK increased markedly over time in both sexes (both BH-FDR *p* < 0.001). In males, the group × time interaction was significant (χ^2^(8) = 45.59, BH-FDR *p* < 0.001). Holm-adjusted comparisons showed that YP was higher than C immediately after EIMD (geometric-mean ratio, 1.800; 95% CI, 1.238–2.617; *p* = 0.029) but lower than C at 48 h (0.524; 95% CI, 0.360–0.761; *p* = 0.011). No other male time-specific comparison remained significant after Holm adjustment. In females, the CK interaction was significant in the outcome-specific analysis (*p* = 0.036) but not after damage-domain BH-FDR correction (q = 0.071). At 24 h, CK was lower in YP than C (ratio, 0.509; 95% CI, 0.350–0.741; Holm-adjusted *p* = 0.006); the remaining female comparisons did not survive Holm adjustment and the female finding was considered exploratory under the broader sensitivity analysis.

In the original-scale CK sensitivity analysis, the immediate male YP–C contrast was not significant. This scale-dependent immediate contrast was therefore not interpreted as evidence that YP increased muscle damage; the primary interpretation focused on the later recovery pattern.

**Figure 2:**
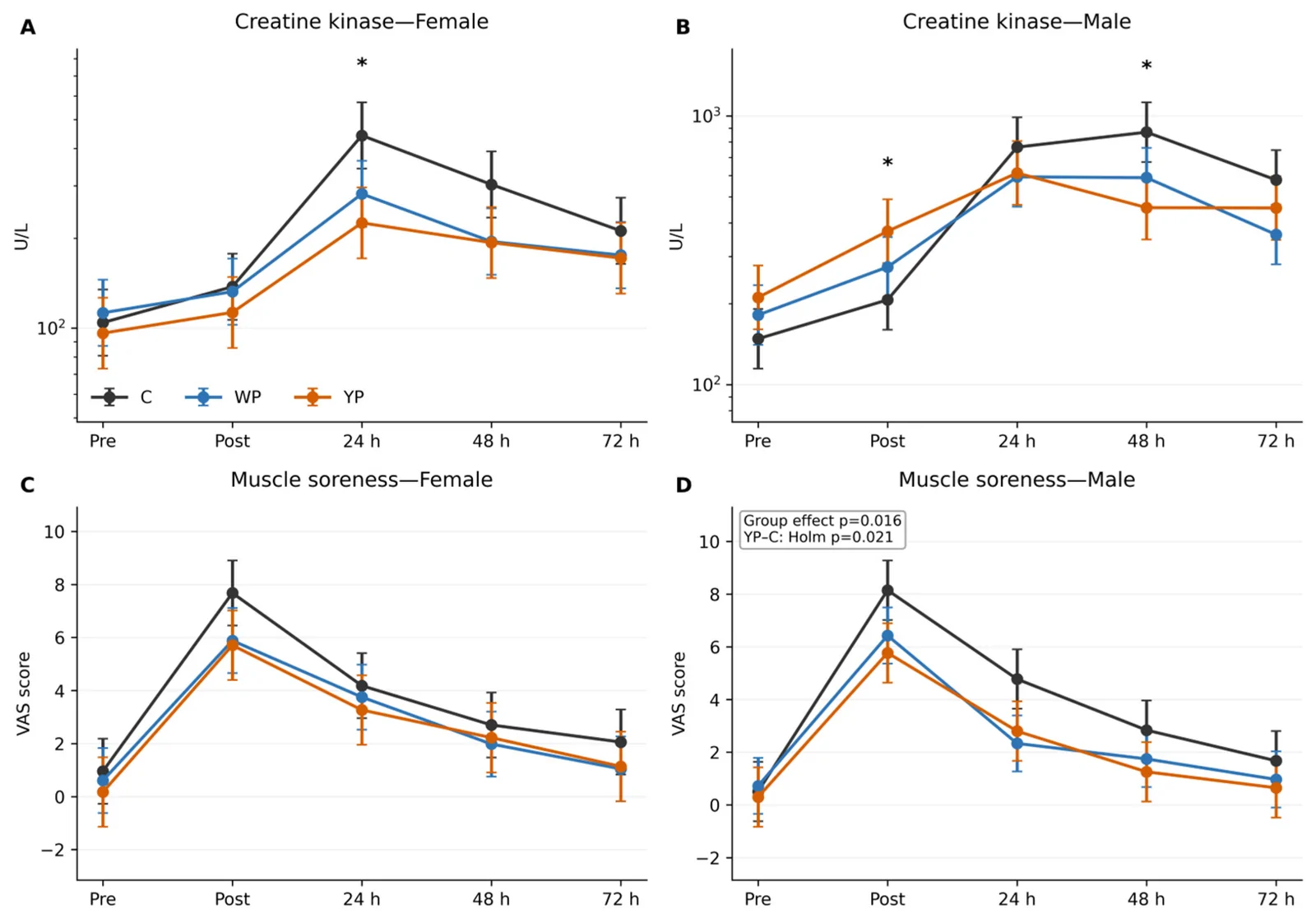
Sex-stratified changes in serum creatine kinase and muscle soreness following EIMD. (**A**) Serum CK in female athletes; (**B**) serum CK in male athletes; (**C**) muscle soreness in female athletes; (**D**) muscle soreness in male athletes. Data are estimated means with 95% CIs; CK values are back-transformed geometric means. Symbols indicate Holm-adjusted comparisons (* YP versus C). The female 24 h CK finding was exploratory after BH-FDR correction; the male CK and VAS findings were retained. C, water control; WP, whey protein; YP, yeast protein–whey protein composite.

Soreness increased after EIMD and declined during follow-up (time effect in both sexes, BH-FDR *p* < 0.001). In males, the interaction was not significant (*p* = 0.195), whereas the group main effect was significant (*p* = 0.016). Averaged across the five assessments, YP had lower VAS than C (mean difference, −1.43 units; 95% CI, −2.47 to −0.39; Holm-adjusted *p* = 0.021). The WP–C comparison did not reach significance after Holm adjustment (−1.15; 95% CI, −2.16 to −0.14; adjusted *p* = 0.051). No group effect was detected in females. Sex-stratified CK and muscle-soreness trajectories are presented in Figure 2.

### 3.3. Inflammatory Responses

**Figure 3:**
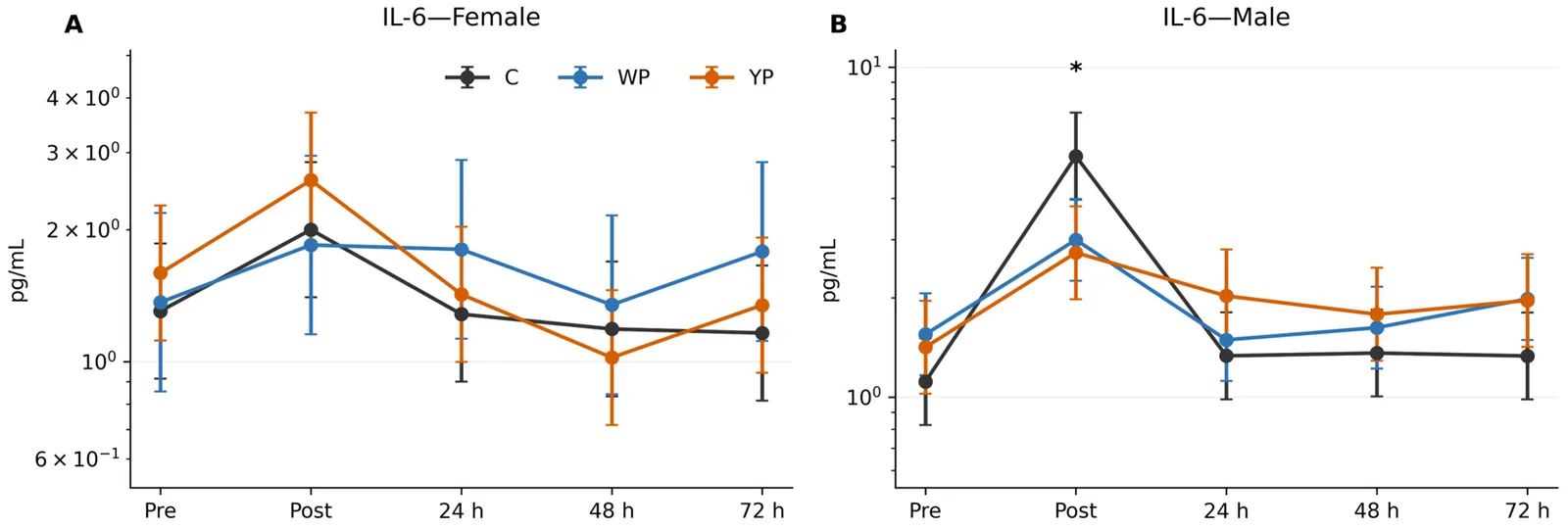
Sex-stratified changes in circulating IL-6 following EIMD. (**A**) Serum IL-6 in female athletes; (**B**) serum IL-6 in male athletes. Data are back-transformed estimated geometric means with 95% CIs. * YP versus C, Holm-adjusted *p* < 0.05. The male group × time interaction was retained after BH-FDR correction. C, water control; WP, whey protein; YP, yeast protein–whey protein composite.

In males, IL-6 showed a significant group × time interaction (χ^2^(8) = 31.14, BH-FDR *p* < 0.001). Immediately after EIMD, IL-6 was lower in YP than C (ratio, 0.510; 95% CI, 0.327–0.795; Holm-adjusted *p* = 0.044); the WP–C comparison did not remain significant after Holm adjustment. The female IL-6 interaction was not significant. Sex-stratified changes in circulating IL-6 are shown in Figure 3. IL-1β, TNF-α, and IL-10 showed no consistent Holm-adjusted intervention-related effects in either sex (Figure S1).

### 3.4. Muscle Function and Performance

**Figure 4:**
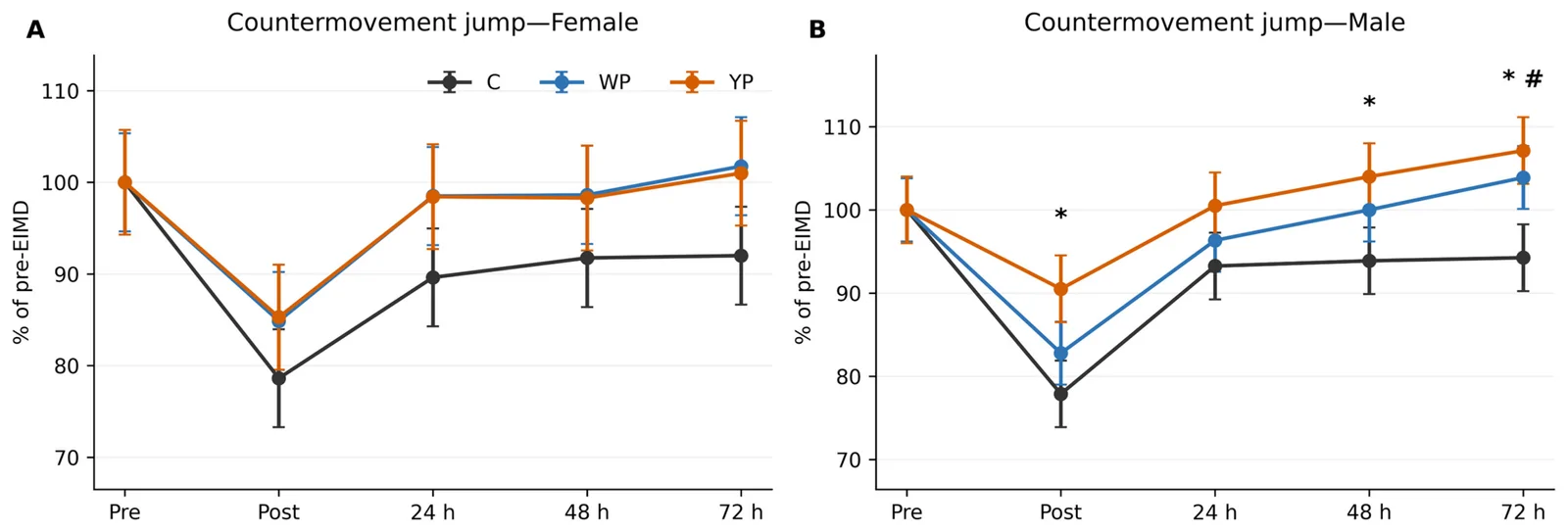
Sex-stratified changes in countermovement-jump performance following EIMD. (**A**) countermovement jump in female athletes; (**B**) countermovement jump in male athletes. Values are expressed relative to baseline (100%) and presented as estimated means with 95% CIs. * YP versus C and # WP versus C, Holm-adjusted *p* < 0.05. The male time-specific findings were exploratory after BH-FDR correction. CMJ, countermovement jump; C, water control; WP, whey protein; YP, yeast protein–whey protein composite.

CMJ declined after EIMD and recovered over 72 h. In males, the group × time interaction was significant in the outcome-specific analysis (*p* = 0.010) but not after performance-domain BH-FDR correction (q = 0.072); the time-specific findings are therefore exploratory. Holm-adjusted contrasts favored YP over C immediately after EIMD (+12.63 percentage points; *p* < 0.001), at 48 h (+10.13; *p* = 0.006), and at 72 h (+12.88; *p* < 0.001); WP exceeded C at 72 h (+9.64; *p* = 0.007). In females, the interaction was not significant (*p* = 0.437), whereas the group main effect was significant (*p* = 0.032). Averaged across all five assessments, the unadjusted differences favored YP over C (+6.20 percentage points; 95% CI, 0.65–11.75; raw *p* = 0.029) and WP over C (+6.35; 95% CI, 0.99–11.71; raw *p* = 0.020), but neither comparison remained significant after Holm adjustment (both adjusted *p* = 0.061). Sex-stratified CMJ trajectories are presented in Figure 4.

**Figure 5:**
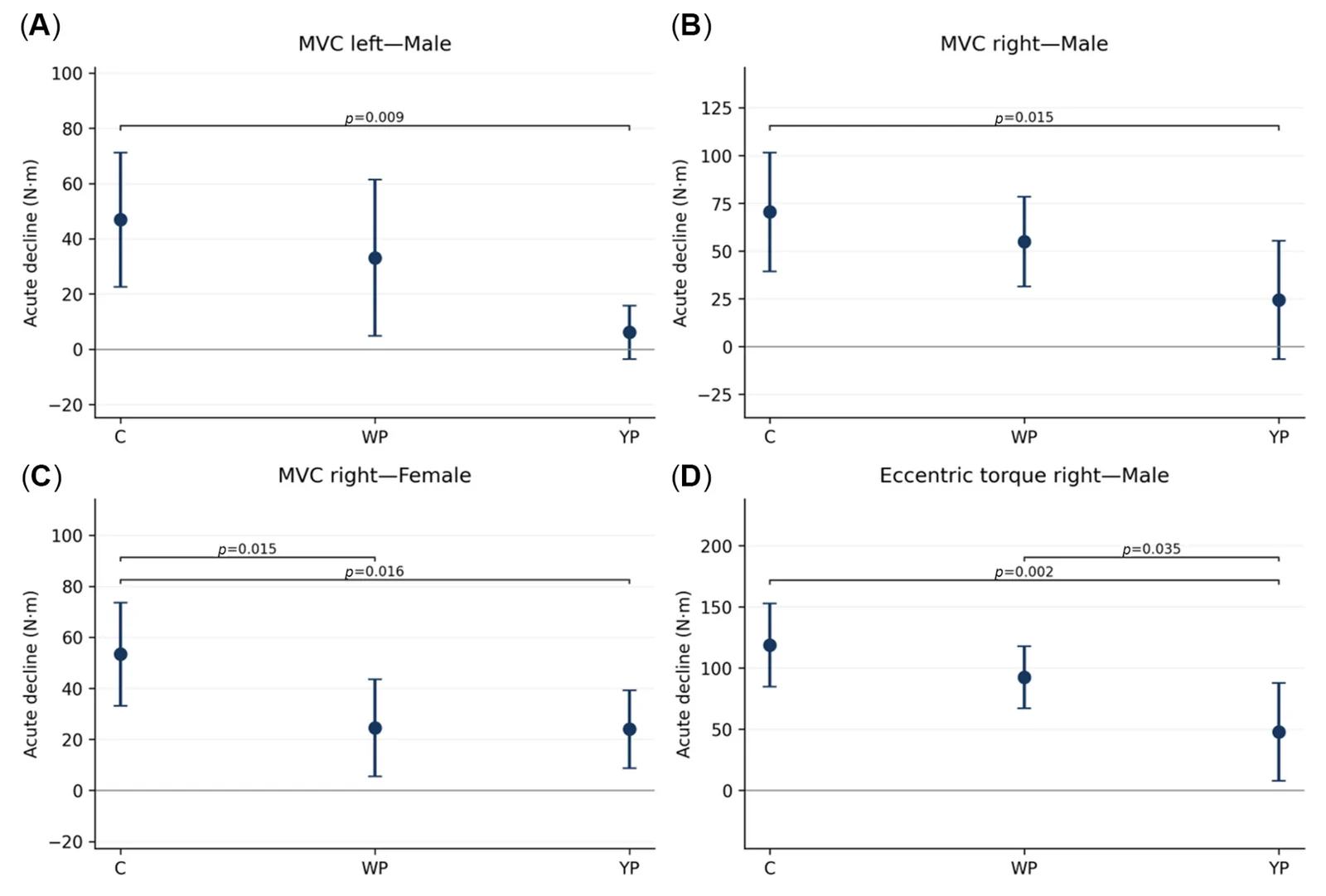
Acute reductions in knee-extensor muscle function immediately following EIMD. (**A**) MVC left in male athletes; (**B**) MVC right in male athletes; (**C**) MVC right in female athletes; (**D**) eccentric torque in male athletes. Larger positive values indicate greater functional loss. Data are means with 95% CIs; brackets indicate Fisher’s protected LSD comparisons. Only the male right-leg eccentric peak-torque YP–C comparison was retained after BH-FDR correction; the other displayed comparisons were exploratory. MVC, maximal voluntary isometric contraction; C, water control; WP, whey protein; YP, yeast protein–whey protein composite.

Acute strength protection was evaluated as the individual decline from baseline to immediately post-EIMD, with a larger positive value indicating a greater loss. In males, the decline differed among groups for left-leg MVC (F(2, 22) = 4.30, *p* = 0.027), right-leg MVC (F(2, 22) = 3.64, *p* = 0.043), and right-leg eccentric peak torque (F(2, 22) = 6.25, *p* = 0.007). Protected LSD comparisons showed smaller declines with YP than C in left-leg MVC (mean difference, 40.75 N·m; 95% CI, 11.38–70.12; *p* = 0.009; Hedges’ g = 1.36), right-leg MVC (46.09 N·m; 95% CI, 9.99–82.20; *p* = 0.015; g = 1.25), and right-leg eccentric peak torque (71.03 N·m; 95% CI, 28.88–113.19; *p* = 0.002; g = 1.65). The right-leg eccentric decline was also smaller in YP than WP (44.52 N·m; 95% CI, 3.55–85.49; *p* = 0.035; g = 1.04). In females, right-leg MVC differed among groups (F(2, 20) = 4.71, *p* = 0.021); both YP (29.38 N·m; 95% CI, 6.00–52.75; *p* = 0.016; g = 1.28) and WP (28.88 N·m; 95% CI, 6.29–51.46; *p* = 0.015; g = 1.26) showed smaller declines than C. Only the male right-leg eccentric YP–C comparison remained significant in the broader BH-FDR sensitivity analysis (q = 0.037). Acute muscle-function declines for outcomes with a significant omnibus group effect are presented in Figure 5.

Exploratory changes from immediately post-EIMD to 24, 48, and 72 h did not provide robust evidence of between-group differences in the rate of MVC or isokinetic-torque recovery. Female right-leg MVC showed a nominal protected-LSD difference for the 24 h gain, but variance homogeneity was violated and the Welch sensitivity test was not significant; this finding was therefore not interpreted as accelerated recovery. The strength findings primarily indicate attenuation of the immediate functional decrement. Complete sex- and limb-specific MVC and isokinetic peak-torque trajectories are presented in Figure S2.

## 4. Discussion

The principal finding of this study was that YP was associated with favorable changes in selected EIMD-related outcomes, particularly in male athletes. The clearest pattern was observed in the male analysis, although the study was not powered to compare intervention effects between female and male athletes. YP was associated with lower CK at selected later recovery time points and lower average soreness across assessments in males, although the male CK trajectory was temporally complex. YP also attenuated the immediate male IL-6 response. Selected CMJ results and acute-decline analyses of MVC and eccentric peak torque suggested greater preservation of muscle function, although most functional findings were exploratory under the broader BH-FDR sensitivity analysis. WP also showed favorable effects relative to water for selected outcomes. Collectively, the findings indicate an outcome-specific response rather than a uniform effect across all biochemical, perceptual, inflammatory, and functional manifestations of EIMD [7,23]. Because the trial registration did not designate a single primary biochemical or functional endpoint, the findings were interpreted using the prespecified longitudinal analyses, with their robustness further evaluated through the broader BH-FDR sensitivity analysis.

CK provided the clearest biochemical evidence of an intervention-related difference, although the pattern was time- and sex-specific. YP was associated with lower CK at 48 h in males and at 24 h in females. The male group × time interaction remained significant after damage-domain BH-FDR correction. In females, however, the group × time interaction did not remain significant after the broader correction; therefore, the Holm-significant 24 h YP–C contrast was interpreted as exploratory. The immediate male YP–C difference was significant in the primary log-transformed analysis but not in the original-scale sensitivity analysis. Circulating CK is an indirect and highly variable systemic marker of sarcolemmal perturbation rather than a direct measure of structural muscle damage [1,24]. The scale-dependent immediate difference was therefore not interpreted as evidence that YP increased muscle damage. Overall, the findings suggest a time-specific biochemical response to YP, particularly the lower male CK value at 48 h, rather than consistently lower muscle damage throughout recovery.

This pattern is broadly consistent with the protein-supplementation literature. A recent systematic review and meta-analysis found that peri-exercise protein supplementation may attenuate CK elevations at later recovery time points, although estimates were heterogeneous [12]. Similarly, in a randomized trial using the same supplemental protein dose of 0.9 g·kg^−1^·day^−1^, whey protein attenuated later increases in CK and myoglobin following eccentric exercise, whereas neither whey protein nor pea protein significantly improved soreness or fitness-test performance [15]. The present findings extend this evidence to a yeast protein–whey protein composite. Nevertheless, because CK is influenced by release kinetics, clearance, training status, sex, and substantial interindividual variability [24], the observed differences cannot be attributed specifically to reduced structural muscle damage. Nor can the study determine whether they resulted from total protein provision, the yeast–whey blend, energy supplied by the formulations, or a yeast-specific constituent. The later CK differences therefore support a possible effect of the complete supplementation strategy on the biochemical recovery response. Because C received water only, the relative contributions of protein provision, energy intake, expectancy, and formulation-specific components remain to be determined.

VAS captured a complementary subjective dimension of recovery. In men, average soreness was lower in YP than C after Holm adjustment, whereas the WP–C comparison was borderline. Because the group × time interaction was not significant, this finding indicates lower soreness averaged across assessments rather than a demonstrably faster recovery trajectory. Because participant blinding to the water-only control was not possible and blinding success was not assessed, expectancy effects cannot be excluded. This outcome-specific interpretation is consistent with systematic reviews showing no consistent overall effect of protein supplementation on post-exercise soreness [12,14].

The lower immediate IL-6 response in male YP suggests modulation of the acute exercise-related cytokine and metabolic response. IL-6 is rapidly released from contracting muscle and has both metabolic and immune functions [25,26]; a lower circulating value should therefore not automatically be interpreted as suppression of harmful inflammation or direct evidence of less structural damage. Inflammation also contributes to debris clearance, repair, and remodeling after damaging exercise [1,8].

IL-1β, TNF-α, and IL-10 did not show consistent intervention-related changes. Thus, the observed cytokine signal was limited to immediate IL-6 in men, without evidence of broader modulation of the measured cytokine profile. Cytokine responses following damaging or resistance exercise vary according to the exercise protocol, sampling time, participant characteristics, and analyte measured, and circulating concentrations do not necessarily reflect the local intramuscular inflammatory response [1,8,27]. Although the cytokines were registered primary outcomes, no single primary inflammatory marker was designated; their findings were interpreted in the context of the broader multiplicity analysis.

CMJ reflects integrated lower-body neuromuscular performance and explosive power. The more favorable CMJ responses observed with YP suggest partial preservation of lower-body performance following EIMD. This may be practically relevant because EIMD can impair jumping, sprinting, and change-of-direction performance for up to 72 h [28,29,30]. A recent meta-analysis also indicated that peri-exercise protein supplementation may reduce strength loss and support the recovery of selected functional outcomes, although findings vary across protocols and populations [12]. Because CMJ depends on multiple muscle groups and neural coordination, the present findings indicate a possible protective effect on overall lower-body performance rather than isolated preservation of knee-extensor function.

The smaller bilateral MVC declines from baseline to immediately post-EIMD in men provide more specific evidence of the partial preservation of isometric knee-extensor strength with YP. This contrast represents protection against the initial functional impairment rather than faster subsequent recovery. EIMD-related force loss may involve impaired force transmission, disturbed calcium regulation, excitation–contraction coupling failure, and structural disruption [29,31,32]. Previous trials have likewise reported attenuation of post-eccentric-exercise force loss or favorable recovery of strength and power with whey-based supplementation [33,34,35,36], supporting the biological plausibility of the present findings.

The isokinetic results were more selective. In men, the smaller immediate reduction in right-leg eccentric peak torque with YP suggests partial preservation of dynamic strength. Protein supplementation has improved isometric or isokinetic force recovery in some studies [37,38], but not others [15,39], potentially because of differences in training status, damaging protocols, habitual protein intake, and supplement composition. The limb-specific findings should be interpreted cautiously because limb dominance was not recorded and testing always proceeded from the right to the left leg. The stronger right-leg signal may reflect limb dominance, order-related fatigue, or familiarization and does not establish a right-limb-specific biological action. Considered together, the CMJ, MVC, and peak-torque findings suggest that YP may help preserve selected aspects of lower-limb strength and power. However, only the male right-leg eccentric YP–C acute-decline contrast remained significant after broader BH-FDR correction; the other functional findings were exploratory, and the recovery-change analyses did not provide robust evidence of accelerated recovery.

The three-arm design addressed two related questions. Comparisons of YP or WP with C assessed whether each complete protein-supplementation strategy modified recovery relative to water, whereas the direct YP–WP comparison assessed whether partial replacement of whey with yeast-derived protein altered the observed response. YP showed favorable differences from water for selected outcomes, and its overall response pattern was generally consistent in direction with WP. This pattern supports the recovery-related activity of the yeast–whey formulation. However, most direct YP–WP comparisons were not significant, and the protected acute-decline contrast favoring YP for right-leg eccentric torque was exploratory after broader BH-FDR correction. Thus, the present findings support partial incorporation of yeast-derived protein as a promising formulation strategy but do not demonstrate superiority over whey protein alone.

Yeast protein is an emerging microbial protein source with substantial protein content and a broad indispensable-amino-acid profile [16,17]. Its nutritional quality, digestibility, nucleic-acid content, functional properties, and sensory characteristics depend on the yeast strain and processing method [16,17,18]. Fermentation enables rapid biomass production and reduces reliance on livestock-derived raw materials, although its environmental and economic performance depends on the specific production process and product [40]. Human studies examining yeast protein supplementation during recovery from EIMD remain scarce. The present findings extend the available evidence by supporting the incorporation of yeast protein as a partial component of a whey-based recovery formulation, although they do not establish a yeast-specific bioactive mechanism. Manufacturer-provided composition data showed that YP and WP were closely matched for protein and calculated energy delivery. Their small differences in carbohydrate and fat content were modest relative to the overall similarity of the two formulations.

Combining yeast and whey may have practical formulation value. Whey provides a rapidly digested, leucine-rich protein with an established role in stimulating post-exercise muscle protein synthesis [9,10], whereas partial blending increases the contribution of microbial protein and may help address the formulation and sensory limitations of yeast protein used alone [17,19,41]. However, amino-acid kinetics, digestibility, muscle protein synthesis, sensory acceptance, cost, and environmental impact were not measured. These factors should therefore be evaluated directly in future formulation-specific studies.

The strengths of this study include its randomized design, assessor-blinded outcome assessment, isonitrogenous comparison of the protein formulations, and multidimensional evaluation of EIMD recovery. The inclusion of well-trained competitive athletes classified in China as first-class or master athletes also enhances the practical relevance of the findings to competitive sports nutrition. Several limitations should be considered. First, although the prespecified sex-stratified analyses allowed intervention responses to be evaluated separately in female and male athletes, the modest group sizes limited the precision of these estimates. The assessment of multiple outcomes and time points also increased the multiplicity burden; accordingly, findings not retained after the broader BH-FDR sensitivity analysis were interpreted as exploratory. Second, the water-only control did not permit participant blinding or control for energy intake, sensory characteristics, and expectancy. Dietary habits were monitored using meal photographs but were not standardized or repeatedly quantified, leaving some potential for residual dietary variation. Third, completed set counts provided only a partial measure of EIMD workload because total mechanical work and time under tension were not recorded. In addition, limb dominance was not recorded, and the fixed right-to-left testing order may have contributed to the limb-specific findings. Finally, the inclusion of athletes from several sports improves the practical breadth of the sample but also introduces variation in habitual loading patterns and training adaptations. The small numbers within several disciplines precluded reliable sport-specific adjustment. These considerations support confirmation of the present findings in larger trials with prespecified functional outcomes and adequate power for direct YP–WP comparisons.

## 5. Conclusions

In well-trained competitive athletes, supplementation with an isonitrogenous yeast protein–whey protein composite favorably influenced selected responses during recovery from EIMD compared with water alone. These effects included lower CK at selected later recovery time points, lower average muscle soreness, a lower immediate post-EIMD IL-6 value, and partial preservation of selected measures of lower-limb strength and power. The clearest pattern was observed in the male analysis, while several functional findings require confirmation under broader multiplicity control. Importantly, the overall response pattern of YP was broadly aligned with that of WP, supporting the recovery-related potential and practical value of incorporating yeast-derived protein into a whey-based formulation.

Although most direct YP–WP comparisons did not indicate an additional advantage of either formulation, the findings support the yeast protein–whey protein composite as a promising protein strategy for promoting selected aspects of recovery between closely scheduled training sessions in competitive athletes.

## Figures and Tables

**Table 1:** Participant characteristics and habitual dietary intake.

Group	Sex	*n*	Age (y)	Height (cm)	Body Mass (kg)	Energy (kcal/day)	Protein (g/day; g/kg/day)
YP	F	7	19.6 ± 0.5	167.1 ± 7.8	59.6 ± 7.6	2058 ± 316	68.9 ± 8.4; 1.17 ± 0.21
WP	F	8	21.1 ± 1.5	175.0 ± 6.5	66.9 ± 9.5	2292 ± 129	75.2 ± 4.5; 1.13 ± 0.09
C	F	8	20.2 ± 1.1	174.6 ± 9.2	64.2 ± 10.2	2315 ± 174	76.1 ± 5.7; 1.20 ± 0.10
YP	M	8	19.0 ± 1.1	184.2 ± 6.6	80.3 ± 6.7	3204 ± 265	104.0 ± 7.9; 1.30 ± 0.05
WP	M	9	20.1 ± 1.7	186.2 ± 9.3	80.2 ± 13.1	3022 ± 359	98.5 ± 12.0; 1.24 ± 0.13
C	M	8	20.9 ± 1.5	186.6 ± 9.8	77.8 ± 9.4	2968 ± 244	97.3 ± 7.7; 1.26 ± 0.06

Values are mean ± SD. F, female; M, male.

## Data Availability

The data presented in this study are available from the corresponding author upon reasonable request. The data are not publicly available because the ethical approval and participant consent did not include unrestricted public sharing of participant-level data.

## Supplementary Materials

The following supporting information can be downloaded at https://www.mdpi.com/article/10.3390/nu18203358/s1, Figure S1: Sex-stratified changes in circulating cytokines following EIMD; Figure S2: Female limb-specific trajectories of maximal voluntary isometric contraction and isokinetic peak torque following exercise-induced muscle damage; Figure S3: Male limb-specific trajectories of maximal voluntary isometric contraction and isokinetic peak torque following exercise-induced muscle damage; Table S1: Composition and indispensable-amino-acid profiles of the finished protein formulations; Table S2: Completed EIMD sets and repetitions by sex and intervention group; Table S3: Outcome-specific sample sizes, omnibus mixed-model tests, and Holm-adjusted comparisons; Table S4: Distribution of sport disciplines among the analyzed participants by intervention group.
