# Supplementary material for: Effects of Yeast Protein–Whey Protein Composite Supplementation on Recovery from Exercise-Induced Muscle Damage in Well-Trained Athletes: A Randomized Controlled Trial
Source: Nutrients. 2026 Oct 14;18(20):3358. doi: 10.3390/nu18203358 (PMC13708182; doi:10.3390/nu18203358)
Supplement: Supplementary file 1 [file nutrients-18-03358-s001.zip › nutrients-4585951-supplementary.pdf]

Supplementary Materials

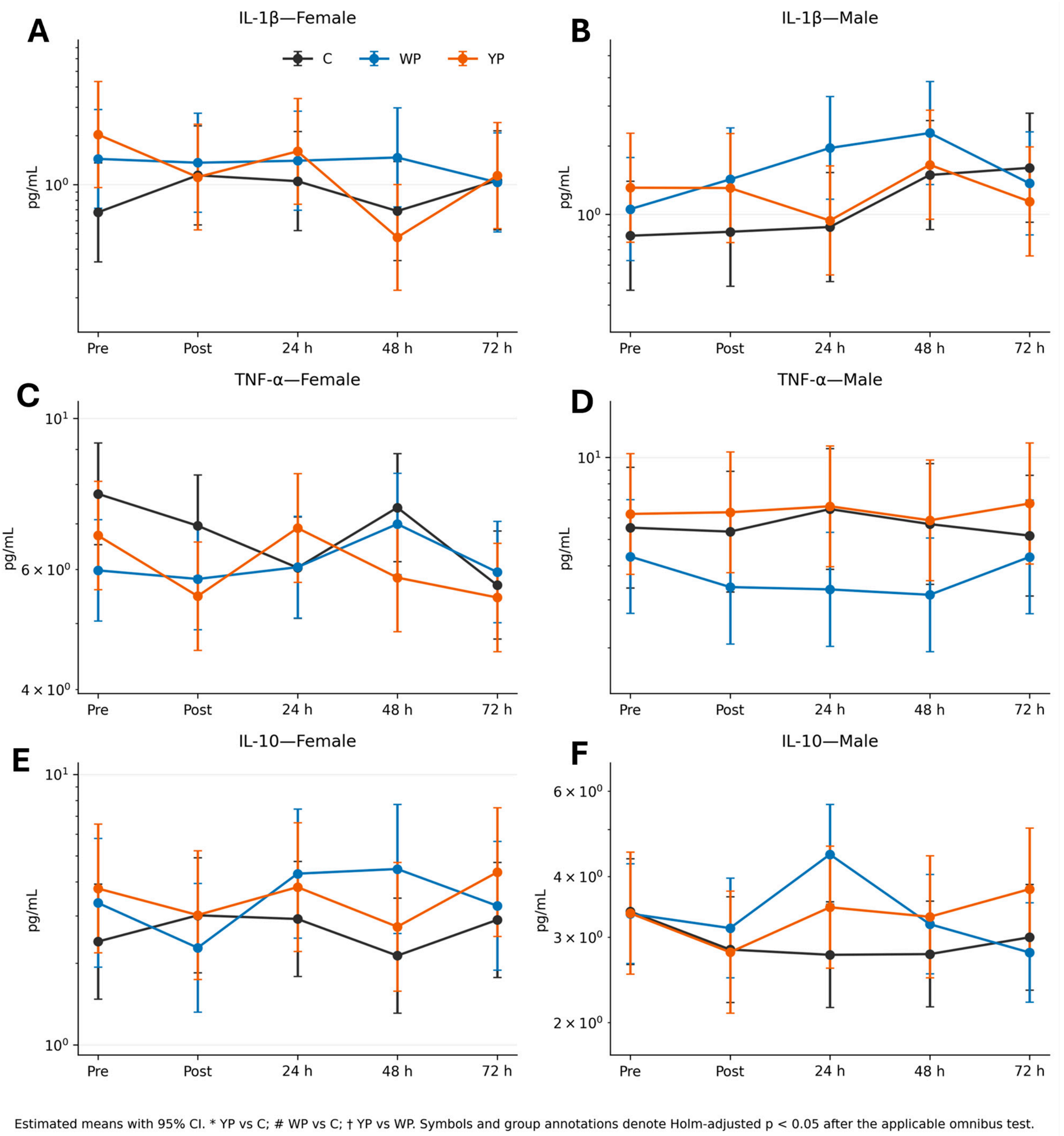

Figure S1. Sex-stratified changes in circulating cytokines following EIMD. (A,B) IL-1β; (C,D) TNF-α; and (E,F) IL-10, presented for female and male athletes, respectively. Data are back-transformed geometric means with 95% CIs. Symbols, if present, denote Holm-adjusted $p < 0.05$; findings not retained after the broader BH-FDR sensitivity analysis are classified as exploratory in Table S1. C, water-only control; WP, whey protein; YP, yeast protein–whey protein composite.

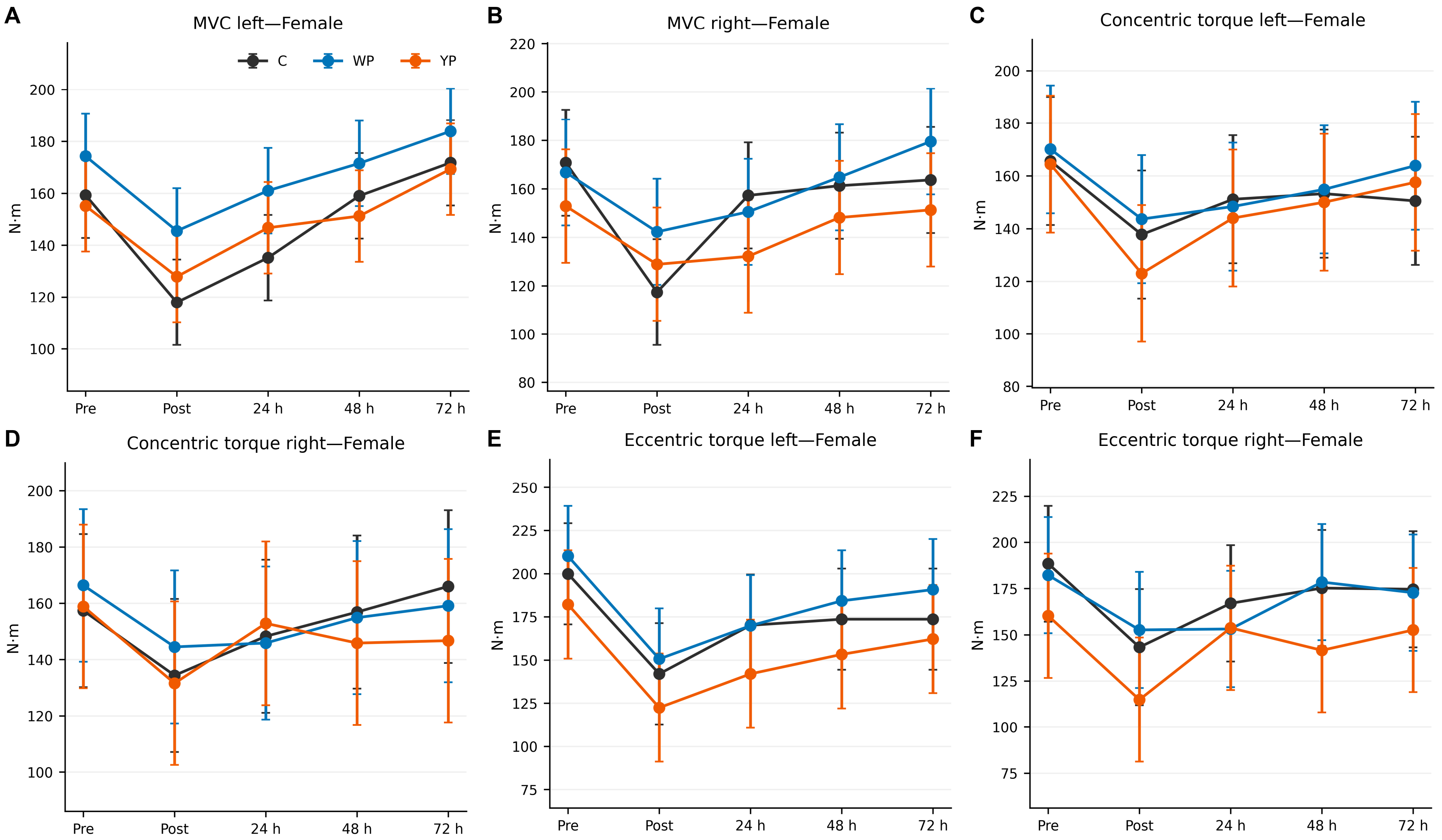

Figure S2. Changes in bilateral knee-extensor muscle function in female athletes following EIMD. (A,B) Left- and right-leg MVC; (C,D) left- and right-leg concentric peak torque; and (E,F) left- and right-leg eccentric peak torque. Data are estimated means with 95% CIs. No displayed longitudinal contrast met the Holm-adjusted criterion; multiplicity results are summarized in Table S1. C, water-only control; WP, whey protein; YP, yeast protein–whey protein composite.

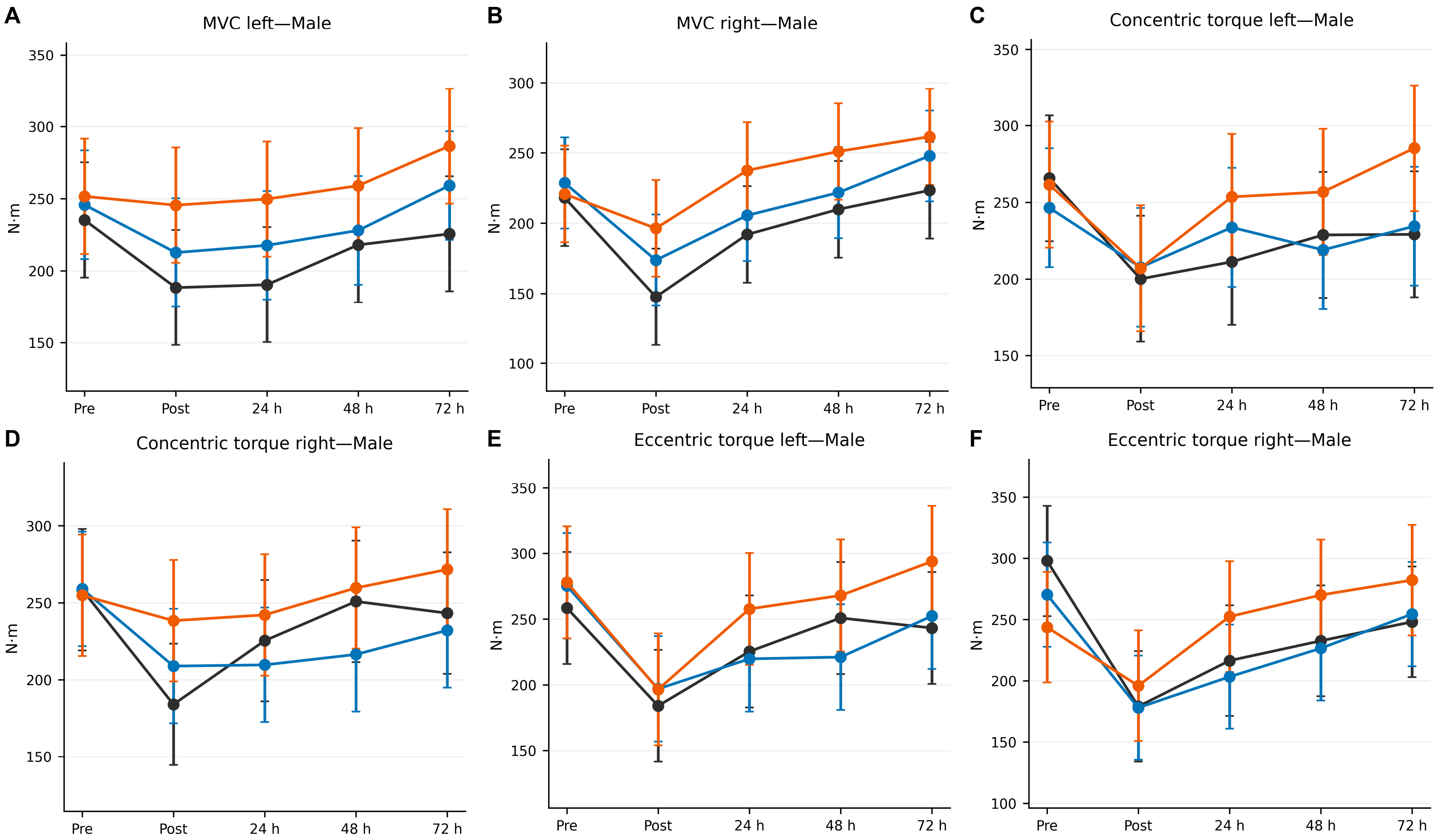

Figure S3. Changes in bilateral knee-extensor muscle function in male athletes following EIMD. (A,B) Left- and right-leg MVC; (C,D) left- and right-leg concentric peak torque; and (E,F) left- and right-leg eccentric peak torque. Data are estimated means with 95% CIs. Longitudinal pairwise significance, when present, was based on Holm-adjusted comparisons; findings not retained after broader BH-FDR correction are classified as exploratory in Table S1. The acute-decline analysis is reported separately in the main manuscript and Figure 5. C, water-only control; WP, whey protein; YP, yeast protein–whey protein composite.

Table S1. Composition and indispensable-amino-acid profiles of the finished protein formulations.

Characteristic	Reporting basis	WP	YP
Energy	kJ/100 g powder	1690	1685
Protein	g/100 g powder	68.9	68.5
Fat	g/100 g powder	5.6	4.3
Carbohydrate	g/100 g powder	18.0	21.2
Sodium	mg/100 g powder	394	269
Nucleic acid	g/100 g powder	ND	0.8
Powder dose per serving	$g\cdot kg^{-1}$ body mass	0.435	0.438
Powder dose per day	$g\cdot kg^{-1}\cdot day^{-1}$	1.306	1.314
Calculated energy provision	$kJ\cdot kg^{-1}\cdot day^{-1}$	22.1	22.1
Isoleucine	g/100 g protein	4.91	6.26
Leucine	g/100 g protein	12.23	12.84
Lysine	g/100 g protein	9.21	10.85
Methionine + cysteine	g/100 g protein	4.84	4.58
Phenylalanine + tyrosine	g/100 g protein	5.73	8.93
Threonine	g/100 g protein	4.08	5.34
Tryptophan	g/100 g protein	1.66	1.80
Valine	g/100 g protein	4.68	10.85
Histidine	g/100 g protein	1.21	2.06
Sum of listed indispensable amino acids	g/100 g protein	48.55	63.51

Values were supplied by the product manufacturer for the finished formulations and were not independently analyzed by the investigators. Energy and proximate composition are expressed per 100 g powder; amino-acid values are expressed per 100 g protein. The sum of listed indispensable amino acids was calculated from the individual values shown. ND, not detected; WP, whey protein; YP, yeast protein–whey protein composite.

Table S2. Completed EIMD sets and repetitions by sex and intervention group.

Sex	Group	n	Left-leg sets	Right-leg sets	Total sets	Total repetitions
Female	YP	7	10.3 ± 0.8 (10–12)	10.0 ± 0.0 (10–10)	20.3 ± 0.8 (20–22)	202.9 ± 7.6 (200–220)
Female	WP	8	10.0 ± 0.0 (10–10)	10.3 ± 0.7 (10–12)	20.3 ± 0.7 (20–22)	202.5 ± 7.1 (200–220)
Female	C	8	10.8 ± 0.9 (10–12)	10.4 ± 0.7 (10–12)	21.1 ± 1.5 (20–24)	211.3 ± 14.6 (200–240)
Male	YP	8	11.4 ± 3.5 (10–20)	10.5 ± 1.4 (10–14)	21.9 ± 4.9 (20–34)	218.8 ± 49.1 (200–340)
Male	WP	9	10.2 ± 0.4 (10–11)	10.2 ± 0.4 (10–11)	20.4 ± 0.9 (20–22)	204.4 ± 8.8 (200–220)
Male	C	8	11.8 ± 3.4 (10–20)	11.4 ± 3.5 (10–20)	23.1 ± 6.9 (20–40)	231.3 ± 68.8 (200–400)

Values are mean ± SD (range). Each set comprised 10 repetitions; total repetitions were calculated as total completed sets × 10. Total set counts did not differ among intervention groups within females (Kruskal–Wallis p = 0.182) or males (p = 0.425). Total mechanical work and time under tension were not recorded. C, water-only control; EIMD, exercise-induced muscle damage; WP, whey protein; YP, yeast protein–whey protein composite.

Table S3. Outcome-specific sample sizes, omnibus mixed-model tests, and Holm-adjusted pairwise comparisons.

Outcome	Sex	Available n/time point (YP/WP/C)	Group×time p [BH q]	Group p [BH q]	Time p [BH q]	Holm-significant contrast, estimate (95% CI), p_Holm
CK	F	7/8/8	0.036 [0.071]	Not interpreted	Not interpreted	24 h: YP vs C, ratio 0.51 (0.35, 0.74), pH=0.006
CK	M	8/9/8	<0.001 [<0.001]	Not interpreted	Not interpreted	Post: YP vs C, ratio 1.80 (1.24, 2.62), pH=0.029; 48 h: YP vs C, ratio 0.52 (0.36, 0.76), pH=0.011
VAS	F	7/8/8	0.874 [0.874]	0.209 [0.209]	<0.001 [<0.001]	None
VAS	M	8/9/8	0.195 [0.195]	0.016 [0.032]	<0.001 [<0.001]	Average: YP vs C, difference -1.43 (-2.47, -0.39), pH=0.021
IL-6	F	7/8/8	0.333 [0.512]	0.729 [0.972]	<0.001 [<0.001]	None
IL-6	M	8/9/8	<0.001 [<0.001]	Not interpreted	Not interpreted	Post: YP vs C, ratio 0.51 (0.33, 0.79), pH=0.044
IL-1β	F	7/8/8	0.398 [0.512]	1.000 [1.000]	0.511 [0.588]	None
IL-1β	M	8/9/8	0.322 [0.429]	0.353 [0.607]	0.041 [0.082]	None
TNF-α	F	7/8/8	0.172 [0.512]	0.293 [0.904]	0.071 [0.142]	None
TNF-α	M	8/9/8	0.491 [0.491]	0.194 [0.607]	0.568 [0.568]	None
IL-10	F	7/8/8	0.512 [0.512]	0.452 [0.904]	0.588 [0.588]	None
IL-10	M	6/9/8	0.200 [0.400]	0.455 [0.607]	0.280 [0.373]	None
CMJ	F	7/8/8	0.437 [0.996]	0.032 [0.193]	<0.001	None

Outcome	Sex	Available n/time point (YP/WP/C)	Group×time p [BH q]	Group p [BH q]	Time p [BH q]	Holm-significant contrast, estimate (95% CI), p_Holm
					[<0.001]	
CMJ	M	8/9/8	0.010 [0.072]	Not interpreted	Not interpreted	Post: YP vs C, difference 12.63 (6.95, 18.30), pH=<0.001; 48 h: YP vs C, difference 10.13 (4.45, 15.80), pH=0.006; 72 h: YP vs C, difference 12.88 (7.20, 18.55), pH=<0.001; 72 h: WP vs C, difference 9.64 (4.12, 15.15), pH=0.007
MVC-L	F	7/8/8	0.841 [0.996]	0.055 [0.193]	<0.001 [<0.001]	None
MVC-L	M	8/9/8	0.350 [0.440]	0.200 [0.539]	<0.001 [<0.001]	None
MVC-R	F	7/8/8	0.124 [0.869]	0.428 [0.599]	<0.001 [<0.001]	None
MVC-R	M	8/9/8	0.480 [0.480]	0.231 [0.539]	<0.001 [<0.001]	None
Concentric torque-L	F	7/8/8	0.882 [0.996]	0.865 [0.922]	<0.001 [<0.001]	None
Concentric torque-L	M	8/9/8	0.187 [0.327]	0.505 [0.589]	<0.001 [<0.001]	None
Concentric torque-R	F	7/8/8	0.725 [0.996]	0.922 [0.922]	<0.001 [<0.001]	None
Concentric torque-R	M	8/9/8	0.085 [0.198]	0.477 [0.589]	<0.001 [<0.001]	None
Eccentric torque-L	F	7/8/8	0.996 [0.996]	0.291 [0.599]	<0.001 [<0.001]	None
Eccentric torque-L	M	8/9/8	0.377 [0.440]	0.493 [0.589]	<0.001 [<0.001]	None
Eccentric torque-R	F	7/8/8	0.782 [0.996]	0.363 [0.599]	<0.001 [<0.001]	None
Eccentric torque-R	M	8/9/8	0.047 [0.166]	Not interpreted	Not interpreted	None

Notes: Available sample sizes are shown in YP/WP/C order; where counts varied by time, each assessment is listed. Raw omnibus p values are followed by BH-FDR q values in brackets. pH denotes the Holm-adjusted pairwise p value. Pairwise contrasts shown in the final column met the prespecified omnibus and Holm criteria. Findings for which the corresponding broader BH-FDR q value was ≥ 0.05 were classified as exploratory in the manuscript. CK and cytokine effects are ratios; other effects are mean differences. Abbreviations: BH-FDR, Benjamini–Hochberg false discovery rate; CI, confidence interval; CK, creatine kinase; CMJ, countermovement jump; MVC, maximal voluntary isometric contraction; C, water-only control; WP, whey protein; YP, yeast protein–whey protein composite.

Table S4. Distribution of sport disciplines among the 48 analyzed participants by intervention group.

Sport discipline	YP, n	WP, n	C, n	Total, n
Volleyball	8	10	9	27
Wushu	3	2	2	7
Football	2	1	1	4
Track and field	1	2	1	4
Ice hockey	1	1	0	2
Rugby	0	1	1	2
Taekwondo	0	0	1	1
Swimming	0	0	1	1
**Total**	**15**	**17**	**16**	**48**

YP, yeast protein–whey protein composite; WP, whey protein; C, water-only control.
